# Evolution of Adaptive Behaviour in Robots by Means of Darwinian Selection

**DOI:** 10.1371/journal.pbio.1000292

**Published:** 2010-01-26

**Authors:** Dario Floreano, Laurent Keller

**Affiliations:** 1 Laboratory of Intelligent Systems, Ecole Polytechnique Fédérale de Lausanne (EPFL), Lausanne, Switzerland; 2 Department of Ecology and Evolution, University of Lausanne, Biophore, Lausanne, Switzerland

Ever since Cicero's *De Natura Deorum ii.34.*, humans have been intrigued by the origin and mechanisms underlying complexity in nature. Darwin suggested that adaptation and complexity could evolve by natural selection acting successively on numerous small, heritable modifications. But is this enough? Here, we describe selected studies of experimental evolution with robots to illustrate how the process of natural selection can lead to the evolution of complex traits such as adaptive behaviours. Just a few hundred generations of selection are sufficient to allow robots to evolve collision-free movement, homing, sophisticated predator versus prey strategies, coadaptation of brains and bodies, cooperation, and even altruism. In all cases this occurred via selection in robots controlled by a simple neural network, which mutated randomly.

Genes do not specify behaviours directly but rather encode molecular products that lead to the development of brains and bodies through which behaviour is expressed. An important task is therefore to understand how adaptive behaviours can evolve by the mere process of natural selection acting on genes that do not directly code for behaviours. A spectacular demonstration of the power of natural selection comes from experiments in the field of evolutionary robotics [1],[2], where scientists have conducted experimental evolution with robots. Evolutionary robotics has also been advocated as a method to automatically generate control systems that are comparatively simpler or more efficient than those engineered with other design methods because the space of solutions explored by evolution can be larger and less constrained than that explored by conventional engineering methods [3]. In this essay we will examine key experiments that illustrate how, for example, robots whose genes are translated into simple neural networks can evolve the ability to navigate, escape predators, coadapt brains and body morphologies, and cooperate. We present mostly—but not only—experimental results performed in our laboratory, which satisfy the following criteria. First, the experiments were at least partly carried out with real robots, allowing us to present a video showing the behaviours of the evolved robots. Second, the robot's neural networks had a simple architecture with no synaptic plasticity, no ontogenetic development, and no detailed modelling of ion channels and spike transmission. Third, the genomes were directly mapped into the neural network (i.e., no gene-to-gene interaction, time-dependent dynamics, or ontogenetic plasticity). By limiting our analysis to these studies we are able to highlight the strength of the process of Darwinian selection in comparable simple systems exposed to different environmental conditions. There have been numerous other studies of experimental evolution performed with computer simulations of behavioural systems. Reviews of these studies can be found in [4]–[6]. Furthermore, artificial evolution has also been applied to disembodied digital organisms living in computer ecosystems, such as Tierra [7] and Avida [8], to address questions related to gene interactions [9], evolution of complexity [10], and mutation rates [11],[12].

## The Principle of Selection in Evolutionary Robotics

The first proposal that Darwinian selection could generate efficient control systems can be attributed to Alan Turing in the 1950s. He suggested that intelligent machines capable of adaptation and learning would be too difficult to conceive by a human designer and could instead be obtained by using an evolutionary process with mutations and selective reproduction [13]. The development of computer algorithms inspired by the process of natural evolution followed shortly after [14]–[16], but the first experiments on the evolution of adaptive behaviours for autonomous robots were done only in the early 1990s [17]–[19], leading to the birth of the field of evolutionary robotics [1],[2].

**Figure 1:**
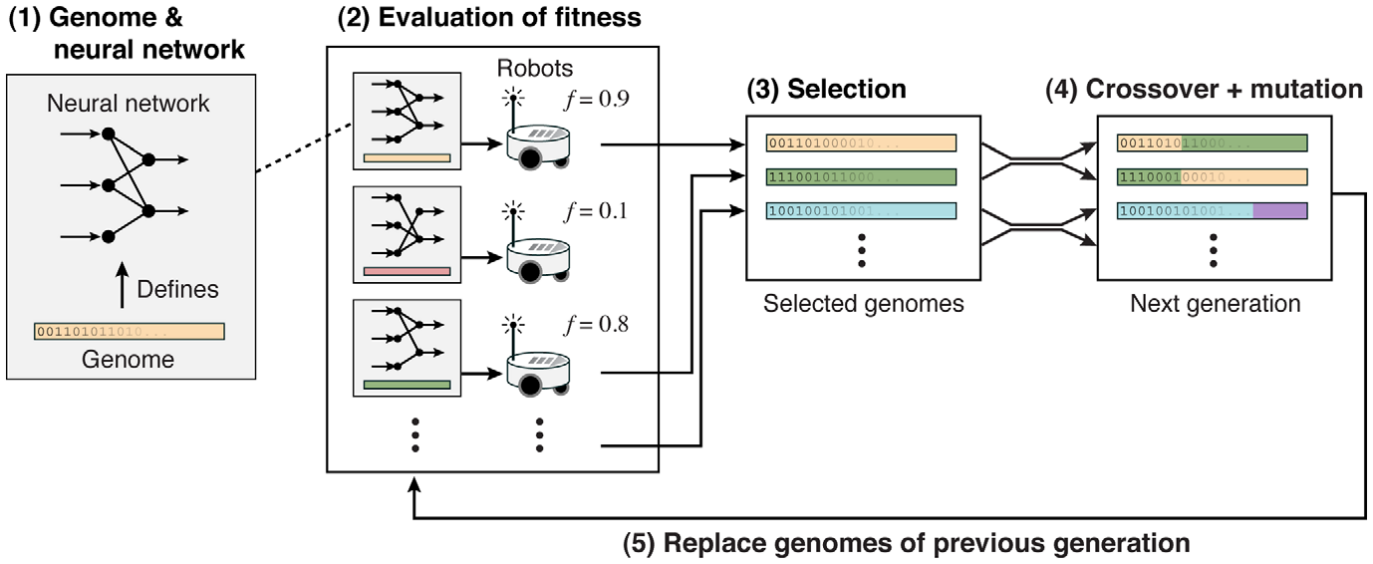
Major steps of Darwinian selection with robots. 1) The robots have a neural network with the strength of connections between neurons determining their behaviour as a function of the information provided by the environment. 2) The fitness *f* of each robot (i.e., the performance in the task assigned to them) is measured in the experimental setting using real robots or physics-based simulators. 3) The genomes of robots with highest fitness are selected to form a new generation. 4) The selected genomes are paired to perform crossover and mutations. 5) The new genomes are used to perform a new round of selection in the next generation.

The general idea of evolutionary robotics (Figure 1 and Video S1) is to create a population with different genomes, each defining parameters of the control system of a robot or of its morphology. The genome is a sequence of characters whose translation into a phenotype can assume various degrees of biological realism [20]. For example, an artificial genome can describe the strength of synaptic connections of an artificial neural network that determines the behaviour of the robot. The input neurons of the neural network are activated by the robot's sensors, and the output neurons control the motors of the robot. Within a population, each individual has a different genome describing a different neural network (i.e., different connections between neurons), thus resulting in specific individual responses to sensory-motor interactions with the environment. These behavioural differences affect the robot's fitness, which is defined, for example, by how fast and straight the robot moves or how frequently it collides with obstacles. At the beginning, robots have random values for their genes, leading to completely random behaviours. The process of Darwinian selection is then imitated by selectively choosing the genomes of robots with highest fitness to produce a new generation of robots. In this process, genomes are paired (to allow recombination) and random mutations (e.g., character substitution, insertion, deletion, or duplication) are applied with a given probability to the new genomes. This process of evolution can be repeated over many generations until a stable behavioural strategy is established. In some experiments this selective process has been performed with real robots whereas in other experiments physics-based simulations [21] that included models of mass, friction, gravity, accelerations, and collisions have been used. Such simulations allow one to conduct selection with a large number of individuals over many generations. The evolved genomes can then be implemented in real robots, which have been shown to display the same behaviour as observed in simulations for the experiments described in this article.

## Collision-Free Navigation

**Figure 2:**
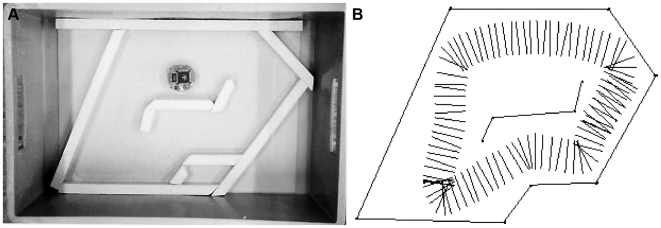
Collision-free navigation. A) A Khepera robot tested in a looping maze. B) Trajectory of one of the robots with an evolved neural controller. The segments represent the axis between the two wheels plotted every 300 ms using an external tracking device.

Darwinian selection has been used to investigate whether small-wheeled robots could evolve collision-free navigation, a behaviour that requires appropriate processing of sensory information and coordinated activation of the motor system. The experiments were conducted in a looping maze (Figure 2, left) with a two-wheeled robot equipped with eight distance sensors (six on one side and two on the other side of the robot). The sensors were connected to eight input neurons that were connected to two output neurons, which each controlled the direction and speed of rotation of one of the wheels (Text S1, section 1). The genome of the robots consisted of a sequence of bits encoding the connection weights between input and output neurons. Mutations allowed the strengths of connections between neurons to change over generations. Experimental selection was conducted in three independent populations each consisting of 80 individuals [18]. The performance of each robot was evaluated with a fitness function describing the ability of the robot to efficiently move in the maze.

Over the first few generations, the robots rapidly improved their ability to move without collisions in the looping maze and, within less than 100 generations, most of them exhibited collision-free navigation (Figure 2, right, and Video S2). Although the fitness function did not specify in what direction the robot should navigate (the robots were perfectly circular and the wheels could rotate in both directions), the best evolved individuals across all replicates moved in the direction corresponding to the side with the highest number of sensors. This was because individuals initially moving in the direction with fewer sensors had higher probability of colliding into corners and thus had lower probability of being selected for reproduction. Interestingly, the driving speed of the best-evolved robots was approximately half of the maximum possible speed and did not increase even when the evolutionary experiments were continued for another 100 generations. Additional experiments where the speed was artificially increased revealed that fast-moving robots had high rates of collisions because the 300-ms refresh rate of the sensors did not allow them to detect walls sufficiently in advance at high speed. Thus, the robots evolved to move at intermediate speeds because of their limited neural and sensory abilities. More generally, these experiments reveal that a process of selective reproduction with random mutations on genes that encode the wiring of neural networks can generate coordinated navigation behaviour that takes into account not only the environmental characteristics, but also the morphological and mechanical properties of the robots.

## Homing

**Figure 3:**
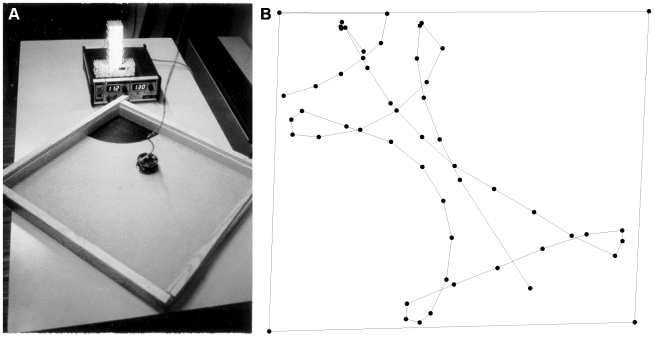
Evolution of homing. A) Experimental setup with a Khepera robot moving in the direction of the nest (recharging station), located in front of the light tower. B) Trajectory of an evolved robot after 200 generations. The trajectory starts in the lower left corner and ends within the recharging nest in the top left corner. Each point corresponds to the recording of the robot's position using an external tracking device. The arena and the recharging nest were plotted by manually positioning the robot along their contours.

An evolutionary experiment with the same robots was conducted to investigate whether they could also evolve the ability to find their way home, a process that has been suggested to require the development of internal representations of the environment [22]–[24]. To mimic a situation selecting for homing ability, robots were placed in a dark room with a small light tower located behind their nest, which consisted of a black patch on the floor in one of the corners of a square arena (Figure 3, left). Robots initially had a fully charged (simulated) battery that discharged linearly over 50 sensory-motor cycles. When a robot passed over the black patch of the nest, its battery was instantaneously recharged. As the experiment lasted 150 sensory-motor cycles, a robot had to return at least twice to the nest to be able to continue moving throughout the whole experiment. In addition to the eight distance sensors used in the collision-free experiments, robots also had a floor-colour sensor, enabling them to determine whether they were in the nest; two light sensors on their sides, allowing them to locate the light tower over their nest (but not sufficient to tell precisely the distance); and a sensor giving information on the battery level (Text S1, section 2).

Experimental selection was conducted in a population of 100 individuals [25]. A robot's fitness was proportional to the average rotational speed of the two wheels and distance from the walls (Text S1, section 2). After 200 generations of selection with real robots, the best individuals performed wide explorations of the arena, returning to the nest only when their batteries had approximately 10% residual energy (Video S3). They stayed in the nest only for the time necessary to turn and exit. This was because being in the nest only permitted small fitness increase as the robots' distance to the walls was very small.

The ability of robots to arrive to the nest when their batteries reached a very low level was mediated by the evolution of a neuronal representation of the environment that enabled them to combine information on their location and battery level to precisely time the homing behaviour (Text S1, section 2). The process of neural representation was reminiscent of “place cells” [26] and of “head-oriented cells” [27] in the rat hippocampus, suggesting that artificial organisms may evolve functionally similar internal representations and behavioural strategies as real organisms to solve tasks requiring the simultaneous processing of different sources of information.

## Predator–Prey Coevolution

**Figure 4:**
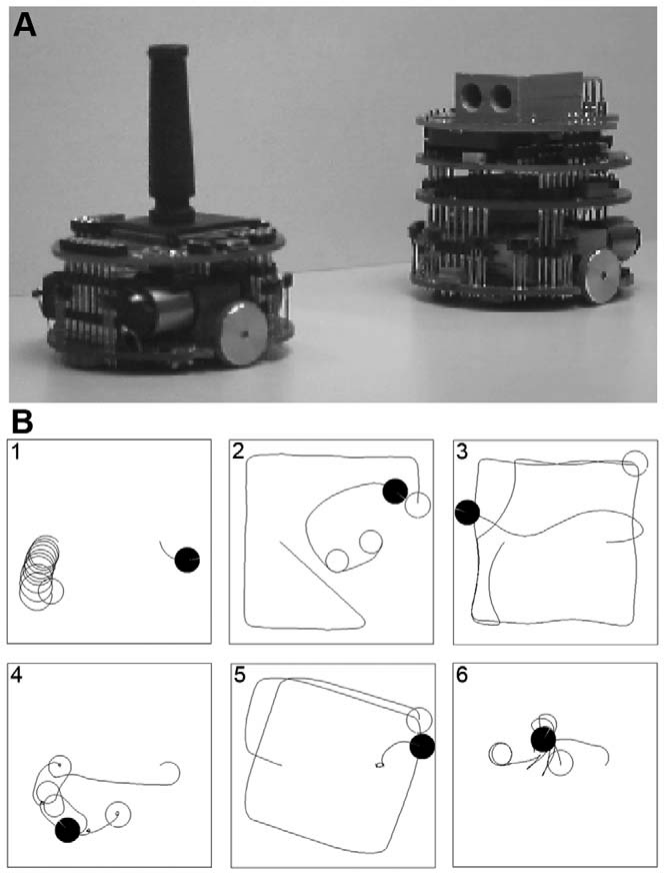
Coevolution of predator and prey robots. A) The predator robot (right) facing the prey robot (left). B) Six examples of pursuit and evasion strategies that evolved over the 100 generations of selection in one of the replicates (see main text for description). The position of the prey at the end of the trial is indicated by the empty disk and that of the predator by the black disk (the lines in the disks correspond to the frontal directions).

Experimental evolution with robots has also been used to study the coevolutionary processes between a population of predator robots and a population of prey robots [28],[29]. Both the predator and prey robots (Figure 4, top) were equipped with eight distance sensors (six on one side and two on the other side). However, prey and predator robots differed in three ways. First, the maximum speed of the prey was twice that of predator. Second, the predator had an additional vision system with a 36° field of view. Third, the prey had a black stick that could be visually perceived by the predator (Text S1, section 3). These differences allowed predators to detect the prey at a distance of up to 100 cm, whereas prey could only detect predators when they were less than 0.5 cm away, but the prey could outrun the predator.

One prey and one predator were placed in pairs in square arenas with the fitness of the predator being inversely proportional to the time it took to catch (i.e., touch) the prey while that of the prey was proportional to the time it managed to avoid being caught by the predator. Each predator robot was individually tested during two minutes against the best prey of each of the previous five generations, and similarly each prey against the five best predators. Starting with populations of 80 predators and 80 prey, each independently tested in one-to-one tournaments, ten independent replicates of 100 generations were carried out in physics-based computer simulations and three replicates of 25 generations were conducted with real robots [30].

Both the simulation and real robot experiments led to the generation of, and cycling through, a set of different pursuit and evasion strategies (Video S4). The cycle observed in one of the simulation replicates is illustrated in Figure 4. During the first generations, most predator and prey robots displayed an uncoordinated behaviour, turning on the spot (Figure 4, box 1). After a few generations, the prey developed fast motion in the environment whereas the predators visually tracked them so as to intercept their trajectories (Figure 4, box 2). Some generations later, the predators became so efficient in catching the prey that they lost the ability to detect and avoid walls (this was due to weak selection pressure for wall avoidance because the prey was almost always caught before the predator would hit a wall) (Figure 4, box 3). Subsequently, the prey evolved a new strategy that consisted of waiting for the predator and moving backward when it approached (Figure 4, box 4), thus avoiding being caught. However, this evasion strategy was not perfect because the prey could not detect the predator when approached from the sides without sensors. A few generations later, the prey displayed a variation of an earlier strategy consisting of coasting the walls at maximum speed. At this point, the predators evolved a “spider” strategy consisting of backing against one of the walls and waiting for the fast-moving prey, whose sensors could not detect the predator sufficiently, early to avoid it because its body reflected less infrared light than the white walls (Figure 4, box 5). After some more generations, the prey displayed a novel variation of the wait-and-avoid strategy where it quickly rotated in place, which reduced the probability of being approached from the sides without sensors. As soon as it detected the predator, it moved backward while facing it with the side having the highest number of sensors (Figure 4, box 6). Overall, these experiments revealed that a large variety of sophisticated behavioural strategies could evolve, but none of them were stable over time because of the coevolutionary dynamics. A similar pattern seems to occur in natural systems where each party in a coevolutionary relationship exerts selective pressure on the other, thereby affecting each other's evolution and leading to a constant evolution of strategies and counterstrategies between parties [31],[32].

## Joint Evolution of Brains and Body Morphologies

Experimental evolution has also been used to coevolve artificial brains and morphologies of simulated robots. In a pioneering study, Karl Sims used a competitive scenario where the fitness of two opponent robots was proportional to their ability to gain control of a cube placed in the centre of an arena [33]. The evolutionary experiments were carried out solely in physics-based simulations. The genome of each robot consisted of two chromosomes, one encoding the topology of a neural network and the other encoding the shape of a body composed of rigid blocks linked by controllable articulations. This led to the coevolution of different types of robots capable of moving towards the cube and preventing access to its opponent. For example, some robots consisted of a cubic block with two articulated, arm-like structures, which were used for moving on the ground and holding the cube. Other robots were composed of only two articulated worm-like segments where one segment was so large and heavy that, once placed over the cube, it prevented the opponent from displacing it.

**Figure 5:**
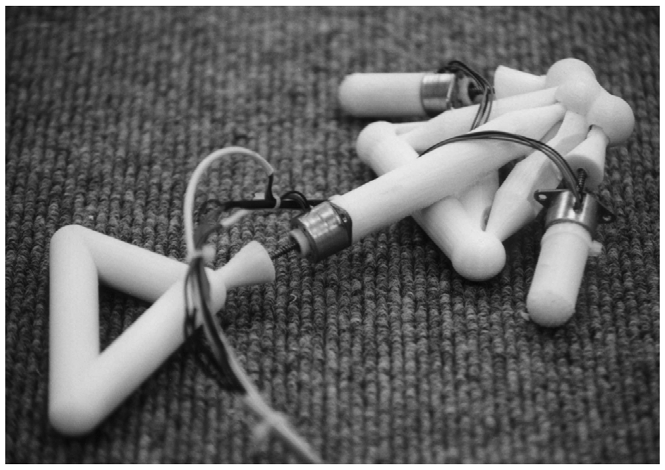
Example of an evolved “creature” created by autonomous design and fabrication process. (Image: Hod Lipson).

The idea of fully evolvable robot hardware was taken on by Lipson and Pollack [34], who applied Darwinian selection to simulated electromechanical systems. As in Sims' experiments, the genome of the evolving individuals specified the morphology of the robot body and of the neural network. The robot bodies consisted of simple building blocks, such as bars of variable lengths, joints, and linear actuators. Bars could be coupled with linear actuators that changed their length and were connected together through ball joints to form arbitrary truss structures with the possibility of both rigid and articulated substructures. The movements of the linear actuators were controlled by the activations of neurons, whose connections to other neurons and to the linear actuators were specified along with the body components in the evolving genomes (Text S1, section 4). The fitness of a robot was proportional to the distance it moved over a flat surface. After 300 generations of selection with physics-based simulations, the individuals with highest fitness were fabricated robotically using rapid manufacturing technology (plastic extrusion 3-D printing) and tested in the real world (Video S5). An example of such a robot capable of fast locomotion is shown in Figure 5. Taken as a whole, these experiments revealed how the coevolution between brain and body morphologies can produce various types of adaptive behaviour and morphologies.

## Evolution of Cooperation and Altruism

**Figure 6:**
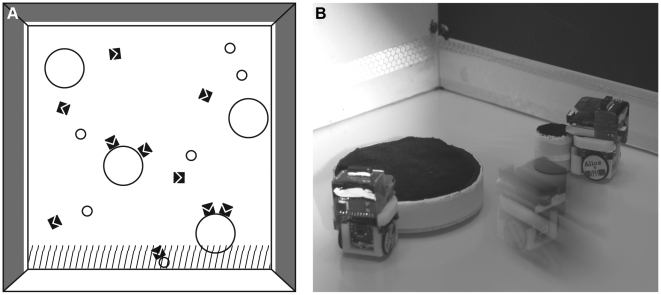
Evolution of cooperative foraging. A) Foraging arena containing ten Alice micro-robots (black squares with arrows) and small and large tokens that robots had to push towards the dashed area near the white wall (the other three walls were painted black). B) Experiment with real robots.

Experimental evolution was also used to investigate whether robots could evolve cooperative and altruistic behaviour and, if so, under what conditions. Cooperation is defined as an act increasing both the direct fitness of the individual giving help and the fitness of the individual receiving help; by contrast, altruism reduces the direct fitness of the individual performing the helping act [35],[36]. The experimental setup consisted of a foraging situation in a square arena containing ten sugar cube-sized wheeled robots, small tokens that a single robot could push, and large tokens requiring at least two robots to be pushed (Figure 6). The robots had five infrared distance sensors, four of them sensing objects within a 3-cm range and a fifth, which was placed higher, having a 6-cm range. These sensors allowed robots to locate the tokens and distinguish them from robots. Robots were also equipped with two vision sensors to perceive the colours of the walls (Text S1, section 5). Their fitness was proportional to the number of tokens successfully pushed within a 4-cm zone along a white wall (the three other walls of the arena were black). A large token successfully pushed along the white wall increased the fitness of all robots within a group (10 robots per group) by 1 fitness unit, while a small token successfully pushed increased the fitness (also by 1 unit) of only the robot that pushed it. The fitness of individual robots was measured in populations containing 100 groups of 10 robots each.

In one experimental condition, the arena contained only large tokens, and the only way for robots to increase their fitness was to cooperate in pushing them [37]. Accordingly, robots readily evolved the ability to cooperatively push large tokens towards the white wall in all 20 evolutionary replicates that were conducted. However, when the arena contained both large and small tokens, the behaviour of robots was influenced by the group kin structure. In groups of unrelated robots (i.e., robots whose genomes where not more similar within than between groups), robots invariably specialised in pushing the small objects, which was the most efficient strategy to maximise their own individual fitness them (i.e., large tokens provided an equal direct payoff as a small token but were more difficult to successfully push). By contrast, the presence of related robots within groups allowed the evolution of altruism. When groups were formed of “clonal” robots all having the same genome, individuals primarily pushed the large tokens even though it was costly, in terms of individual fitness, for the robots pushing (Video S6).

Similar results were obtained in experiments where groups of light-emitting, foraging robots could communicate the position of a food source at a cost to themselves because of the resulting increased competition near food. In these experiments, robots again readily evolved costly communication when they were genetically related, but altruistic communication never evolved in groups of unrelated robots when selection operated at the individual level [38],[39].

These experiments are interesting in two ways. First, they demonstrate that the same general rules apply for experimental evolution of robots and real organisms. Theory predicts that altruism, defined as an act of helping that decreases the direct fitness of the individual performing it, should only evolve among related individuals, and this is also what has been found in a wide range of organisms, ranging from bacteria to social insects and social vertebrates (e.g., [40]–[45]). Second, it demonstrates that cooperation and altruism can evolve even in organisms with simple cognitive abilities (in both the token pushing and communication experiments, robots had neural network controllers consisting of less than 15 neurons).

## 

### Conclusions

These examples of experimental evolution with robots verify the power of evolution by mutation, recombination, and natural selection. In all cases, robots initially exhibited completely uncoordinated behaviour because their genomes had random values. However, a few hundreds of generations of random mutations and selective reproduction were sufficient to promote the evolution of efficient behaviours in a wide range of environmental conditions. The ability of robots to orientate, escape predators, and even cooperate is particularly remarkable given that they had deliberately simple genotypes directly mapped into the connection weights of neural networks comprising only a few dozen neurons.

So far, evolutionary robotic experiments have been conducted mostly by computer scientists and engineers (e.g., [17],[46]–[56]). Their primary interest has been to exploit the power of artificial evolution to automatically generate novel or better control systems and body shapes for specific problems. For example, the method of evolutionary robotics described in the context of cooperative behaviour has been successfully used to generate the control systems of a swarm of micro aerial vehicles that must locate rescuers and spread so as to establish a radio communication network based uniquely on signal strength of the rescuer mobile phones and of the robot emitters, a problem for which existing engineering solutions require the use of absolute geo-localisation information provided by GPS signals [57].

A major issue in evolutionary robotics is that agents may use idiosyncratic features of the environment in which they are selected to increase performance, hence leading to a major fitness drop in new environments where these features are lacking. A similar problem arises when the evolutionary process takes place in simulations failing to capture relevant physical aspects of the environment. In this case, the evolved individuals do not operate well in the real world [58],[59]. Computer scientists and engineers have come up with various solutions to this problem (for a recent review, see [4]). One consists of measuring the fitness of evolving individuals in several environments that vary along relevant dimensions (e.g., lighting conditions or ground texture). Another consists of incorporating noise in features of the simulation model (e.g., elasticity of joints or the physical interactions that occur during collisions) that may not faithfully reflect the real world. A third consists of coevolving the robot and the key parameters of the simulation model and periodically testing the evolved control system with real robots to improve the estimate of the fitness of the robot and simulator [53]. Finally, a solution that may also be relevant from a biological perspective consists of adding ontogenetic plasticity to the evolving individuals so that they can adapt to environmental modifications arising during their lifetime [60].

It is only very recently that biologists and cognitive scientists have become interested in evolutionary robotics, realising that it provides a powerful means to study how phenotypes can be shaped by natural selection and address questions that are difficult to address with real organisms. Current topics of biologically motivated research in evolutionary robotics include the role of ontogenetic development (e.g., [61]), the principles of neural control of highly dynamic and elastic body morphologies such as passive robotic walkers (e.g., [62],[63]), the functional role of morphology in coevolving bodies and brains [64], the role of active perception as a mean to structure and simplify sensory information in behaving organisms [65],[66], and the effects of synaptic plasticity [60],[67],[68] and neuromodulation [69] on organisms evolving in rapidly changing and partially unpredictable environments (i.e., under situations where individuals benefit to change behaviour over time). In particular, the incorporation of adaptive mechanisms during ontogeny mediated by phenotypic plasticity and learning (e.g., [70]) provides promising avenues for the study of processes operating at different spatial and temporal scales.

In comparison to theoretical and numerical models of biological phenomena, the embodiment and behavioural features of robot models can result in stronger testing of hypotheses and in higher predictive power [71]–[73]. The use of real robot features are particularly useful in an evolutionary perspective where behaviour and ensuing complex physical interactions can significantly affect the interaction with the environment and performance. Therefore, evolutionary robotics also offers new opportunities to address issues such as sexual selection, division of labour, speciation, and, in general, the open-ended evolution of diversity and complexity in behavioural systems. Interdisciplinary collaborations among engineers, evolutionary biologists, neuroscientists, and molecular biologists should prove fruitful to investigate important issues on the principles that mediate the evolution of adaptive behaviour of organisms that cannot be readily studied with standard methods.

## Supporting Information

Text S1 **Supplementary methods.**
(6.50 MB DOC)

Video S1 **Method for evolving the neural network of a robot.** Valid gene sequences are extracted (magnifying lens) from a binary string representing the genome of the robot. Those genes are translated into neurons of different type (colour) according to the genetic specifications, such as sensory, motor, excitatory, or inhibitory neurons. The corresponding neural network is connected to the sensors and motors of the robot and the resulting behaviour of the robot is measured according to the fitness function. The genomes of the individuals that had the worst performance are discarded from the population (symbolically thrown in a dustbin) whereas the genomes of the best individuals are paired and crossed over with small random mutations to generate new offspring (the process of selective reproduction is symbolically shown to occur in a “mother robot”). After several generations of selective reproductions with mutations, robots display better or novel behaviours.
(7.82 MB MOV)

Video S2 **Evolution of collision-free navigation.** In the initial generations, robots can hardly avoid walls (one robot even approaches objects). After 50 generations, robots can navigate around the looping maze without hitting the walls.
(8.15 MB MOV)

Video S3 **Evolved “Khepera” robot performing exploration and homing for battery recharge.** The robot enters the recharging area approximately 2 seconds before full battery discharge.
(9.23 MB MPG)

Video S4 **Coevolved predator and prey robots engaged in a tournament.** After locating and moving towards the prey, the predator cannot reach it because the prey can perceive it with the rear distance sensors and moves faster.
(1.89 MB MOV)

Video S5 **Coevolution of body and brain in a robotic machine.** Please switch the audio on to listen to the commentary. Video courtesy of Hod Lipson, also available from http://www.mae.cornell.edu/Lipson/.
(4.97 MB MOV)

Video S6 **Evolution of altruistic cooperation in a team of clonal “Alice” robots.** In the initial generation, the robots can hardly perform coordinated navigation. After 240 generations of Darwinian selection, most robots search for large food tokens and cooperate to push them towards the region of the arena under the white wall.
(10.21 MB MPG)

## References

[1] Cliff D, Husbands P, Harvey I (1993). Explorations in evolutionary robotics.. Adapt Behav.

[2] Nolfi S, Floreano D (2000). Evolutionary robotics: the biology, intelligence, and technology of self-organizing machines.

[3] Miller J. F, Job D, Vassilev V. K (2000). Principles in the evolutionary design of digital circuits – part I.. Genetic Programming and Evolvable Machines.

[4] Floreano D, Husbands P, Nolfi S, Siciliano B, Khatib O (2008). Evolutionary Robotics.. Springer handbook of robotics.

[5] Floreano D, Mattiussi C (2008). Bio-inspired artificial intelligence: theories, methods, and technologies.

[6] Harvey I, Di Paolo E, Wood R, Quinn M, Tuci E (2005). Evolutionary robotics: a new scientific tool for studying cognition.. Artif Life.

[7] Ray T. S, Langton C. G, Taylor C, Farmer D. J, Rasmussen S (1992). An approach to the synthesis of life.. Proceedings of the Second Workshop on Artificial Life.

[8] Adami C (1998). Introduction to artificial life.

[9] Lenski R. E, Ofria C, Collier T. C, Adami C (1999). Genome complexity, robustness, and genetic interactions in digital organisms.. Nature.

[10] Lenski R. E, Ofria C, Pennock R. T, Adami C (2003). The evolutionary origin of complex features.. Nature.

[11] Wilke C. O, Wang J, Ofria C, Lenski R. E, Adami C (2001). Evolution of digital organisms at high mutation rate leads to survival of the flattest.. Nature.

[12] Clune J, Misevic D, Ofria C, Lenski R. E, Elena S. F (2008). Natural selection fails to optimize mutation rates for long-term adaptation on rugged fitness landscapes.. PLoS Computational Biology.

[13] Turing A. M (1950). Computing machinery and intelligence.. Mind.

[14] Rechenberg I (1965). Cybernetic solution path of an experimental problem.. Royal Air Force Establishment.

[15] Fogel L. J, Owens A. J, Walsh M. J (1966). Artificial intelligence through simulated evolution.

[16] Holland J. H (1975). Adaptation in natural and artificial systems: an introductory analysis with applications to biology, control, and artificial intelligence.

[17] Lewis M. A, Fagg A. H, Solidum A (1992). Genetic programming approach to the construction of a neural network for control of a walking robot..

[18] Floreano D, Mondada F, Cliff D, Husbands P, Meyer J. A, Wilson S (1994). Automatic creation of an autonomous agent: genetic evolution of a neural network driven robot.. Proceedings of third International Conference on Simulation of Adaptive Behavior: From Animals to Animats 3.

[19] Harvey I, Husbands P, Cliff D, Cliff D, Husbands P, Meyer J. A, Wilson S (1994). Seeing the light: artificial evolution, real vision.. Proceedings of the third International Conference on Simulation of Adaptive Behavior: From Animals to Animats 3.

[20] Floreano D, Dürr P, Mattiussi C (2008). Neuroevolution: from architectures to learning.. Evol Intelligence.

[21] Featherstone R (2000). Robot dynamics: equations and algorithms..

[22] Schmajuk N. A, Blair H. T (1993). Place learning and the dynamics of spatial navigation: A neural network approach.. Adapt Behav.

[23] Burgess N, Donnett J. G, Jeffery K. J, O'Keefe J (1997). Robotic and neuronal simulation of hippocampal navigation.. Philos Trans R Soc Lond B Biol Sci.

[24] Healy S (1998). Spatial representations in animals.

[25] Floreano D, Mondada F (1996). Evolution of homing navigation in a real mobile robot.. IEEE Trans Syst Man Cybern B Cybern.

[26] O'Keefe J, Nadel L (1978). The hippocampus as a cognitive map.

[27] Taube J. S, Muller R. U, Ranck J. B. J (1990). Head-direction cells recorded from the postsubiculum in freely moving rats. I. Description and quantitative analysis.. J Neurosci.

[28] Floreano D, Nolfi S, Koza J. R, Deb K, Dorigo M, Fogel D. B, Garzon M (1997). God save the red queen! Competition in co-evolutionary robotics.. Genetic Programming 1997: Proceedings of the Second Annual Conference.

[29] Nolfi S, Floreano D (1998). Co-evolving predator and prey robots: do “arm races” arise in artificial evolution.. Artif Life.

[30] Floreano D, Nolfi S, Mondada F, Pfeifer R (1998). Competitive co-evolutionary robotics: from theory to practice.. Proceedings of the fifth International Conference on Simulation of Adaptive Behavior: From Animals to Animats 5.

[31] Van Valen L (1973). A new evolutionary law.. Evol Theory.

[32] Bell G (1982). The masterpiece of nature: the evolution and genetics of sexuality.

[33] Sims K, Brooks R, Maes P (1994). Evolving 3D morphology and behavior by competition.. Artificial Life IV.

[34] Lipson H, Pollack J. B (2000). Automatic design and manufacture of robotic lifeforms.. Nature.

[35] Hamilton W. D (1964). The genetical evolution of social behaviour.. J Theor Biol.

[36] Lehmann L, Keller L (2006). The evolution of cooperation and altruism: A general framework and a classification of models.. J Evol Biol.

[37] Waibel M, Keller L, Floreano D (2009). Genetic team composition and level of selection in the evolution of cooperation.. IEEE Trans Evol Comput.

[38] Floreano D, Mitri S, Magnenat S, Keller L (2007). Evolutionary conditions for the emergence of communication in robots.. Curr Biol.

[39] Mitri S, Floreano D, Keller L (2009). The evolution of information suppression in communicating robots with conflicting interests.. PNAS.

[40] Sherman P. W (1977). Nepotism and the evolution of alarm calls.. Science.

[41] Sundström L, Chapuisat M, Keller L (1996). Conditional manipulation of sex ratios by ant workers: A test of kin selection theory.. Science.

[42] Pfennig D. W, Collins J. P (1993). Kinship affects morphogenesis in cannibalistic salamanders.. Nature.

[43] Langer P, Hogendoorn K, Keller L (2004). Tug-of-war over reproduction in a social bee.. Nature.

[44] West S. A, Murray M. G, Machado C. A, Griffin A. S, Herre E. A (2001). Testing Hamilton's rule with competition between relatives.. Nature.

[45] West S. A, Diggle S. P, Buckling A, Gardner A, Griffin A. S (2007). The social lives of microbes.. Annu Rev Ecol Evol Syst.

[46] Gallagher J. C, Beer R. D, Espenschied K. S, Quinn R. D (1996). Application of evolved locomotion controllers to a hexapod robot.. Rob Auton Syst.

[47] Gomi T, Ide K, Sugisaka M (1998). Emergence of gaits of a legged robot by collaboration through evolution.. Proceedings of the International Symposium on Artificial Life and Robotics.

[48] Gruau F, Quatramaran K, Husbands P, Harvey I (1996). Cellular encoding for interactive evolutionary robotics.. Fourth European Conference on Artificial Life.

[49] Kodjabachian J, Meyer J-A (1998). Evolution and development of neural controllers for locomotion, gradient-following, and obstacle-avoidance in artificial insects.. IEEE Trans Neural Netw.

[50] Hornby G. S, Lipson H, Pollack J. B (2003). Generative representations for the automated design of modular physical robots.. IEEE Trans Rob Autom.

[51] Quinn M, Smith L, Mayley G, Husbands P (2003). Evolving controllers for a homogeneous system of physical robots: structured cooperation with minimal sensors.. Philos Trans Phys Sci Eng.

[52] Dorigo M, Trianni V, Sahin E, Groß R, Labella T. H (2004). Evolving self-organizing behaviors for a swarm-bot.. Auton Robots.

[53] Bongard J, Zykov V, Lipson H (2006). Resilient machines through continuous self-modeling.. Science.

[54] Trianni V, Nolfi S, Dorigo M (2006). Cooperative hole avoidance in a swarm-bot.. Rob Auton Syst.

[55] Tellez R, Angulo C, Pardo D, Ijspeert A. J, Masuzawa T, Kusumoto S (2006). Evolving the walking behaviour of a 12 DOF quadruped using a distributed neural architecture.. Proceedings of the International Workshop on Biologically Inspired Approaches to Advanced Information Technology.

[56] Watson R. A, Ficici S. G, Pollack J. B (2002). Embodied evolution: distributing an evolutionary algorithm in a population of robots.. Robotics and Autonomous Systems.

[57] Hauert S, Zufferey J-C, Floreano D (2009). Reverse-engineering of artificially evolved controllers for swarms of robots..

[58] Brooks R. A, Varela F. J, Bourgine P (1992). Artificial life and real robots.. Toward a Practice of Autonomous Systems: Proceedings of the First European Conference on Artificial Life.

[59] Jakobi N, Husbands P, Harvey I, Moran F, Moreno A, Merelo J, Chacon P (1995). Noise and the reality gap: The use of simulation in evolutionary robotics.. Advances in Artificial Life: Proceedings of the Third European Conference on Artificial Life.

[60] Urzelai J, Floreano D (2001). Evolution of adaptive synapses: robots with fast adaptive behavior in new environments.. Evol Comput.

[61] Bongard J, Pfeifer R, Hara F, Pfeifer R (2003). Evolving complete agents using artificial ontogeny.. Morpho-functional Machines: The New Species: Designing Embodied Intelligence.

[62] Reil T, Husbands P (2002). Evolution of central pattern generators for bipedal walking in a real-time physics environment.. IEEE Trans Evol Comput.

[63] Vaughan E. D, Di Paolo E, Harvey I. R, Pollack J, Bedau M, Husbands P, Ikegami T, Watson R (2004). The evolution of control and adaptation in a 3D powered passive dynamic walker.. Proceedings of the Ninth International Conference on the Simulation and Synthesis of Living Systems.

[64] Pfeifer R, Bongard J. C (2006). How the body shapes the way we think.

[65] Bianco R, Nolfi S (2004). Evolving the neural controller for a robotic arm able to grasp objects on the basis of tactile sensors.. Adapt Behav.

[66] Suzuki M, Floreano D, Di Paolo E (2005). The contribution of active body movement to visual development in evolutionary robots.. Neural Netw.

[67] Di Paolo E. A (2003). Evolving spike-timing-dependent plasticity for single-trial learning in robots.. Philos Trans Phys Sci Eng.

[68] Niv Y, Joel D, Meilijson I, Ruppin E (2002). Evolution of reinforcement learning in uncertain environments: A simple explanation for complex foraging behaviors.. Adapt Behav.

[69] Philippides A, Husbands P, Smith T, O'Shea M (2005). Flexible couplings: diffusing neuromodulators and adaptive robotics.. Artif Life.

[70] Parisi D, Nolfi S, Mukesh J. P, Honavar V, Balakrishan K (2001). Developmental in neural networks.. Advances in Evolutionary Synthesis of Neural Networks.

[71] Ijspeert A. J, Crespi A, Ryczko D, Cabelguen J-M (2007). From swimming to walking with a salamander robot driven by a spinal cord model.. Science.

[72] Pfeifer R, Lungarella M, Iida F (2007). Self-organization, embodiment, and biologically inspired robotics.. Science.

[73] Webb B (2002). Robots in invertebrate neuroscience.. Nature.

