# Supplementary material for: Evolution of Adaptive Behaviour in Robots by Means of Darwinian Selection
Source: PLoS Biol. 2010 Jan 26;8(1):e1000292. doi: 10.1371/journal.pbio.1000292 (PMC2811146; doi:10.1371/journal.pbio.1000292)
Supplement: Text S1 — Supplementary methods. (6.50 MB DOC) [file pbio.1000292.s001.doc]

Text S1. Supplementary Methods.

S1: Collision-free navigation

Miniature (6 cm diameter) Khepera robots [1] were equipped with eight infrared distance sensors and two motorized wheels (Fig. S1, top). The robot was controlled by a neural network with eight sensory neurons, each assigned to one of the eight infrared distance sensors and two output motor neurons receiving weighted inputs from each of the eight sensory neurons and from the other output neuron, plus a recurrent connection with itself with a 300 ms delay (Fig. S1, bottom). The net input of an output neuron was summed to the threshold value of the output neuron and the total sum was passed through the logistic squashing function $\frac{1}{1-e^{-x}}$. The resulting outputs, in the range [0,1], were used to control the rotation of the two wheels so that an output of 1 generated maximum rotation speed (8 cm/s) in one direction, an output of 0 generated maximum rotation speed (8 cm/s) in the opposite direction, and an output of 0.5 did not generate any motion in the corresponding wheel. The sensor values and wheel speeds of the robots were refreshed every 300 ms (one sensory-motor cycle).

The genome encoded the weights of the connections between neurons (2 x 9 + 2 = 20 connections) and of the thresholds (2) as binary numbers with eight bits for each value (22 times 8 = 176 genes).

The performance (“fitness”) of a robot was computed over 80 cycles of 300 ms as

$$\Phi=V\left(1-\sqrt{\Delta v}\right)(1-i)$$

where *V* is the average rotation speeds (range [0,1]) of the two wheels independently of their direction, $\Delta v$ is the absolute value of the difference between the directional rotation speeds of the two wheels (speeds in the range [0,1] correspond to rotation in one direction and speeds in the range [-1,0] correspond to rotation in the other direction) and *i* is the value in the range [0,1] of the distance sensor with the highest activity. The first component is maximized by higher rotational speed of the two wheels, the second component by straight motion in one direction or in the other, and the third component by larger distance from objects. The fitness values were accumulated at each sensory-motor cycles and the resulting number was divided by the number of cycles (80) at the end of the evaluation. A fitness value of 1.0 would correspond to a robot moving straight at maximum speed in an open space and therefore was not attainable in the looping maze since some of the distance sensors were always active due to wall proximity and because several turns were necessary to complete tour of the maze.


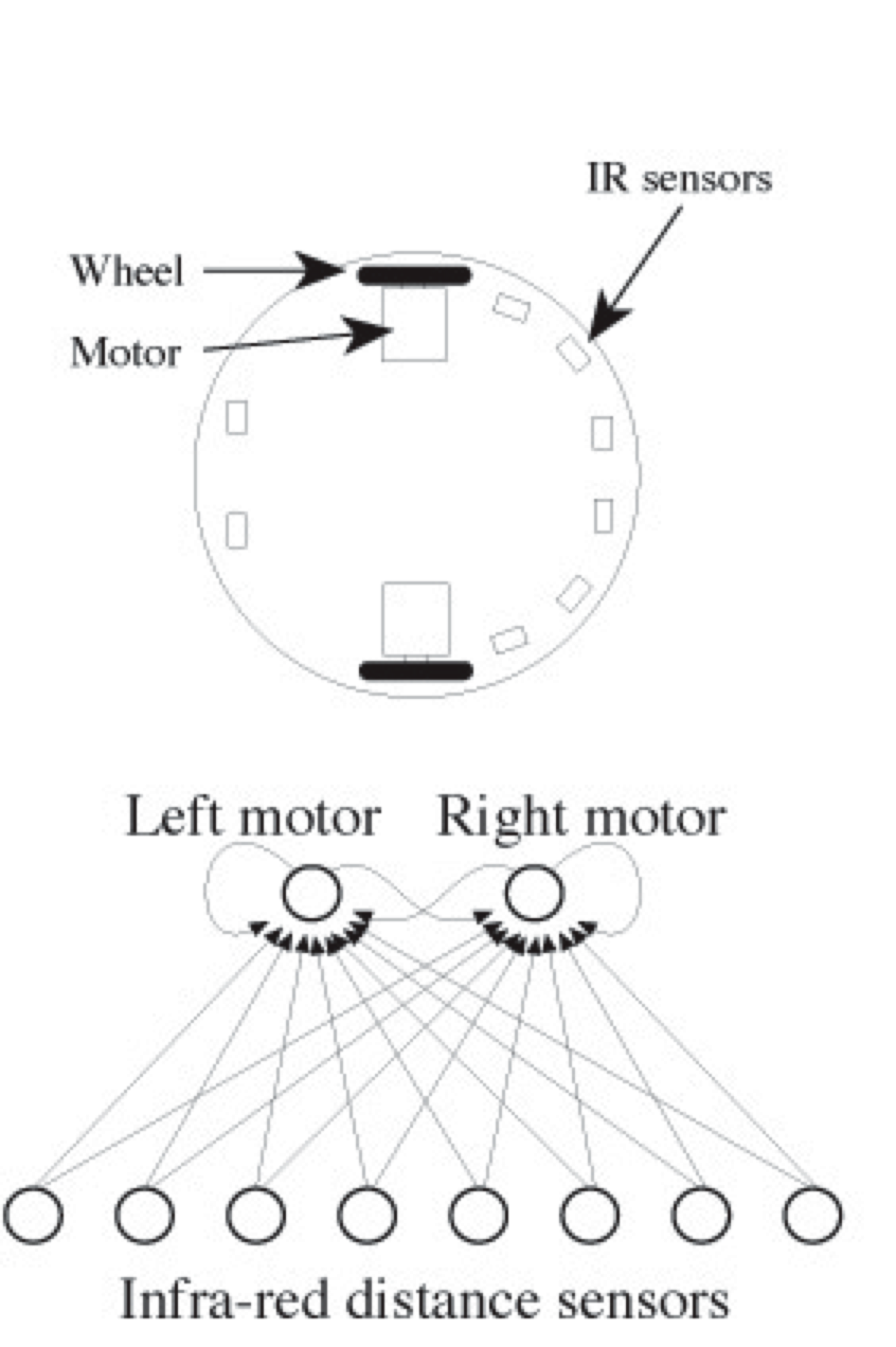


Figure S1. Top: Layout of the sensory-motor components of the Khepera robot used in the collision-free navigation experiments. Bottom: Layout of the neural network.

S2: Homing

Khepera robots were equipped with an additional floor-colour sensor under the body of the robot and with a simulated battery that lasted 50 sensory-motor cycles (Fig. S2, top). Furthermore, two infrared sensors were used to measure also the ambient light in addition to the distance from obstacles. The robot was controlled by a neural network with twelve sensory neurons each assigned to one of the sensors (eight infrared distance sensors, two light sensors, one floor-colour sensor, and one battery sensor). The neural network had five internal units, each receiving weighted inputs from the twelve sensory neurons and from the other four internal units, plus a recurrent connection with itself with a 300 ms delay. Each of the two output neurons of the neural network received weighted inputs from the five internal neurons (Fig. S2, bottom). The net input of each internal and output neuron was summed to its own threshold value and the total sum was passed through the logistic squashing function $\frac{1}{1-e^{-x}}$. The resulting values of the output units, in the range [0,1], were used to control the rotation of the two wheels so that an output of 1 generated maximum rotation speed in one direction, an output of 0 generated maximum rotation speed in the opposite direction, and an output of 0.5 did not generate any motion in the corresponding wheel. The sensor values and wheel speeds of the robots were refreshed every 300 ms (i.e., every sensory-motor cycle).

The genome encoded the individual weights of all the connections ((5 x 16 + 5) + (2 x 5 + 2) = 97 connections) and of the thresholds (5 + 2 = 7 thresholds) as binary numbers with eight bits for each value (104 times 8 = 832 genes).

The fitness of a robot was computed every 300 ms using a simplified version of the function described in S1,

$$\Phi=V(1-i)$$

The fitness values were accumulated over 150 sensory-motor cycles. Whenever robots returned to the nest the battery was instantaneously recharged allowing the robot to live for 50 sensory-motor cycles. Thus, a higher fitness was achieved by robots having a high average wheel speed *V*, maintaining larger distance from walls (1-*i*) and periodically returning to the nest to recharge their battery.


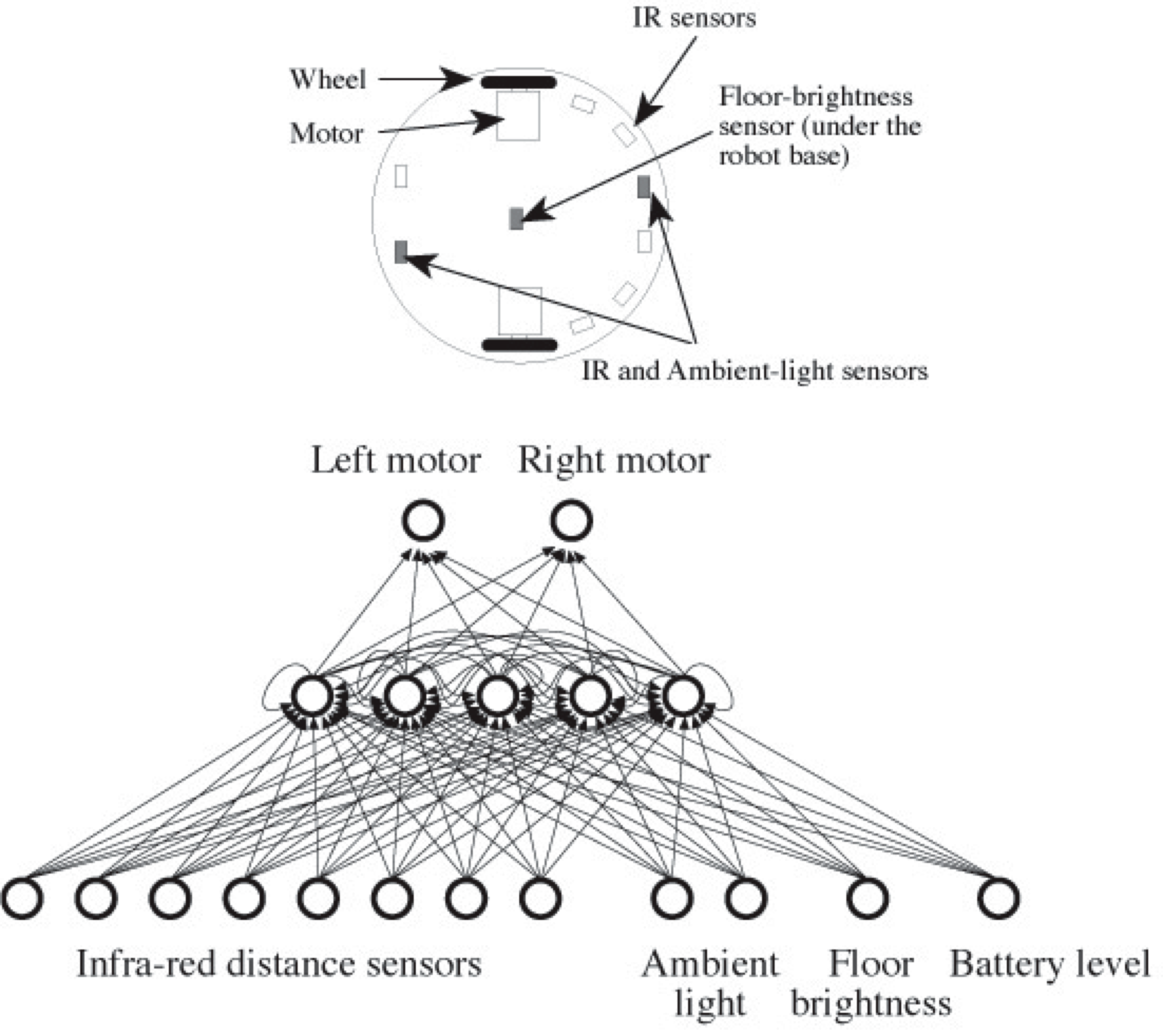


Figure S2. Top: Layout of the sensory-motor components of the Khepera robot used in the exploration and homing experiments. Bottom: Layout of the neural network.

S3: Prey-Predator Co-evolution

Two Khepera robots were used to play the roles of a prey and a predator. Each robot was fitted with a conductive metallic ring at the base in order to detect when they hit each other. The prey robot was equipped with only eight infrared distance sensors, as described in S1. The predator was equipped with the eight infrared distance sensors and with a vision system made of a line of 64 photoreceptors spanning a visual field of 36 degrees (Fig. S3). The neural network of the prey was composed of eight sensory neurons each assigned to one of the eight distance sensors and of two output neurons with recurrent connections to control the direction and speed of rotation of each wheel, exactly as described in S1. The neural network of the predator, in addition to the eight distance sensors, included five sensory neurons whose activation levels were proportional to the average grey levels measured by the photoreceptors in each of the corresponding five visual sectors that divided up the visual field in equal parts. The sensor values and wheel speeds of the robots were refreshed every 100 ms (i.e., every sensory-motor cycle).

The genome of the prey and of the predator encoded the connection weights of the corresponding neural networks. The prey genome was composed of 90 genes (2 motor units x 8 distance sensor units + 2 thresholds = 18 values x 5 bits) and the genome of the predator was composed of 140 genes (2 motor units x (8 distance sensor units + 5 visual sensor units) + 2 thresholds = 28 values x 5 bits).

The fitness of the prey was proportional to the number of sensory-motor cycles spent without being hit by the predator divided by the total number of sensory-motor cycles (1200 sensory-motor cycles of 100 ms in 2 minutes). The fitness of the predator was inversely proportional to the number of sensory-motor cycles spent before hitting the prey (i.e., 1 – the prey fitness). The fitness of an individual was the average value of the five tournaments performed with the best opponents of the last five generations.


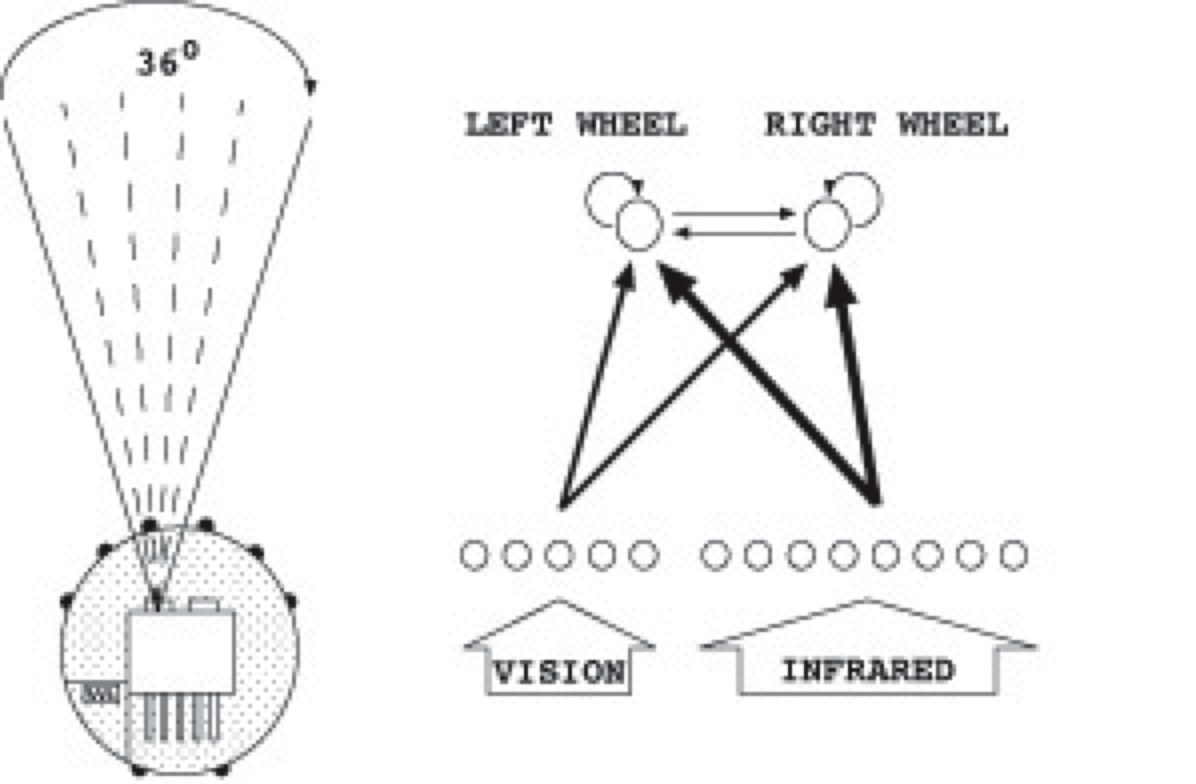


Figure S3. Left: Layout of the vision module of the predator robot used in the prey-predator co-evolution experiments. Right: Layout of the neural network of the predator. In these experiments, the layouts of the prey robot and of the prey neural network are identical to those depicted in Figure S1.

S4: Joint evolution of brains and morphologies

Robots were made of plastic bars connected to each other by ball-and-socket joints (Fig. S4). Some bars were coupled to a motorized linear actuator, which could modify the length of the bar, which resulted in some movement of the robot. The actuation of the motorized bars was controlled by the activation of a corresponding neuron in the co-evolving neural network. The neural network was composed of two or more neurons fully connected to each other through weighted links. In addition, each neuron had its own threshold value. The activation of a neuron was given by the sum of the weighted inputs from the other neurons with its own threshold. Some neurons could be connected to an actuated bar. Since the robot had no sensor, the activation of the neurons, and consequently the motion of the robot, was generated only by the internally-generated activation of the neural network. The states of the robot actuators and of the neuronal activations were synchronously updated at discrete times steps (cycles).

The genome of the robots consisted in a string of integer and real-valued numbers describing properties of vertices (x, y, z), bars (vertex index 1, vertex index 2, extended length, stiffness), neurons (threshold, connection weights to all other neurons), and actuators (bar index, neuron index, and actuation range). Mutations could alter the genome by adding a bar, duplicating an existing gene sequence, or altering the properties of the components. Evolutionary experiments started with a population of 200 empty genomes, whose mutations and selection of the fittest could result in the gradual construction of sophisticated robot and neuronal morphologies (bottom of Fig. S4).

The fitness function was the Euclidian distance that the centre of mass of an individual moved within a predefined number of cycles. Evolutionary experiments were carried out in a simulator that assumed quasi-static motion in which each step of the motion generation was assumed to be statically stable. This assumption facilitated the transfer of the evolved robots from simulation to reality and allowed for motion modalities that had little momentum, such as crawling and walking. Evolved robots that displayed efficient motion were converted into physical machines in three steps. First, the lines and points described in the genome were automatically translated by software into solid models with ball joints and space for incorporating the motors. In the second step, the solid model description was sent to a commercially available rapid-prototyping machine ("3D printer") consisting of a heated head depositing thermoplastic material on a plane layer by layer. In the third step, motors were snapped into the actuated bar slots and the neural network was downloaded into a digital micro-controller to activate the motors. The automatically fabricated robots could faithfully reproduce the motion patterns of their simulated counterparts.


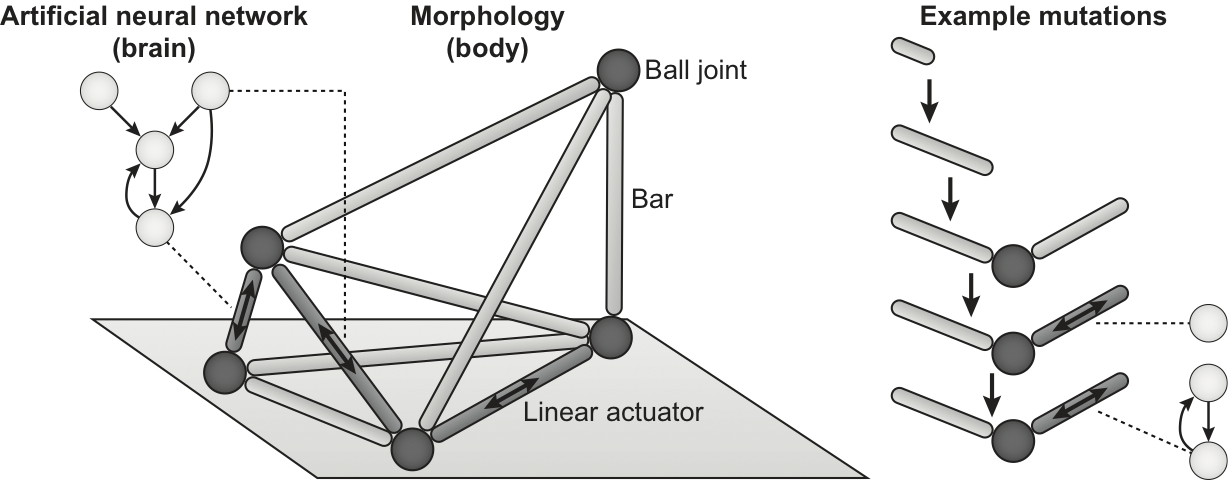


Figure S4. Left: Layout of a co-evolving robot morphology and neural network. Right: Example of a possible sequence of mutations that lead to the creation of a robot. Initially a small bar is created; the bar is elongated and then duplicated; the duplicated bar is mutated into an actuated bar and a neuron appears; finally, a second neuron with reciprocal connections to the previous neuron is added.

S5: Evolution of cooperation and altruism

Ten (2×2×4 cm) Alice robots [2] were placed in foraging arena. Each robot (Fig S5, left) was equipped with two motorized wheels, with three infrared distance sensors at the front and one at the back that could sense objects up to 3 cm away. In addition, an extension turret was mounted on top of the robot containing a fifth infrared distance sensor with a range of up to 6 cm and a vision system made of a line of photoreceptors spanning a visual field of 150 degrees. Only two virtual visual sensors were used as inputs of the neural network, each sensor representing the average of three equi-distally spread photoreceptors over an 18-degree field of view to the left and to the right sides of the image.

The robots were controlled by a neural network with seven sensory neurons each assigned to one of the sensors (five infrared distance sensors and two visual sensors). The neural network had three internal units, each receiving weighted inputs from the seven sensory neurons. Each of the two output neurons of the neural network received weighted inputs from the three internal units (Fig. S5, right). The net input of each internal and output neuron was summed to its own threshold value and the total sum was passed through the squashing function tanh(*x*). The resulting values of the output units, in the range [-1,1], were mapped into speeds in the range [-4; 4] cm/s, with speeds in the interval of [-2.5; 2.5] cm/s set to 0 because of unreliable motor response at low speeds. The sensor and wheel speed values were refreshed every 100 ms (one sensory-motor cycle).

The genome encoded the individual weights of all the connections ((3 x 7) + (2 x 3) = 27 connections) and of the thresholds (3 + 2 = 5 thresholds) as binary numbers with eight bits for each value (32 times 8 = 256 genes).

In the cooperative evolution experiment the arena contained only 4 large tokens and the fitness of all robots in a group was proportional to the number of tokens pushed to the white wall (i.e., the fitness of all robots in a group increased by 1 unit when a large token was successfully pushed). In the altruistic experiment the arena contained 6 small and 4 large tokens and the large token successfully pushed also increased the fitness of all group members by one unit. However, the small token successfully pushed to the white wall provided fitness returns (also 1 fitness unit) only to the robot pushing it. Since small token were much easier to push and because they provided the same reward to the focal individuals as the large token, pushing large token was altruistic (i.e., it led to lower direct fitness for individuals performing the act but higher fitness for other group members [3]). 300 generations of selection were performed in populations consisting of 100 groups of 10 robots. Twenty independent selection experiments were performed for both the cooperative and altruistic conditions. The fitness of robots in all groups was evaluated 10 times for three minutes with tokens and robots being randomly placed at the beginning of each replicate. The fitness was averaged over the 10 replicates.

Because selection was performed over many generation in several independent replicates, all experiments were conducted using a physics-based simulator modelling robot size, morphology, mass, speed, as well as collision dynamics, friction forces and sensor and actuator responses. Evolved individuals that obtained the highest fitness in the evolutionary experiments were transferred to the physical robots to validate the results (see accompanying video 6).


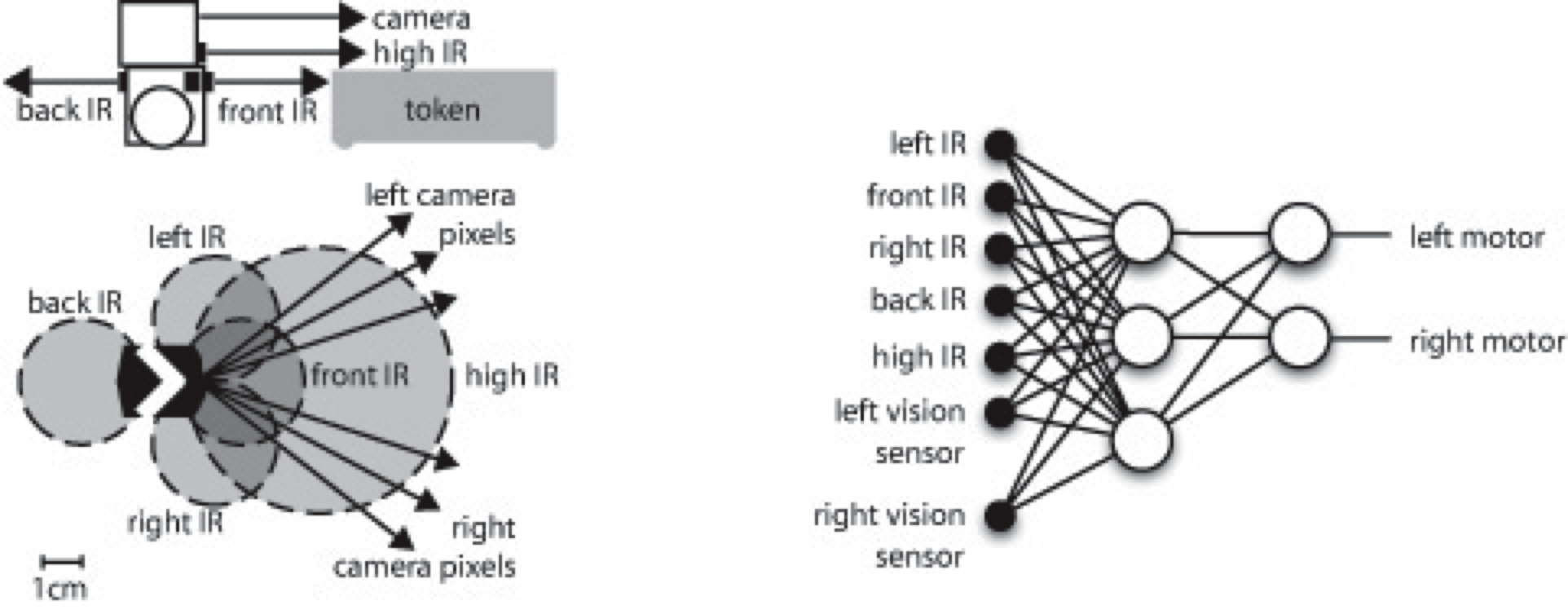


Figure S5. Left: Layout of the sensory-motor components of the Alice robot used in the cooperation and altruism experiments. Left: Layout of the neural network.

**References**

1. Mondada F, Franzi E, Ienne P (1993) Mobile robot miniaturization: A tool for investigation in control algorithms. In: Yoshikawa T, Miyazaki F, editors. Proceedings of the Third International Symposium on Experimental Robotics. Berlin: Springer-Verlag. pp. 501–513.

2. Caprari G, Estier T, Siegwart R (2002) Fascination of Down Scaling - Alice the Sugar Cube Robot. Journal of Micromechatronics 1: 177-189.

3. Lehmann L, Keller L (2006) The evolution of cooperation and altruism: A general framework and a classification of models. J Evol Biol 19: 1365-1379.
